# Malaria and Age Variably but Critically Control Hepcidin Throughout Childhood in Kenya

**DOI:** 10.1016/j.ebiom.2015.08.016

**Published:** 2015-08-08

**Authors:** Sarah H. Atkinson, Sophie M. Uyoga, Andrew E. Armitage, Shivani Khandwala, Cleopatra K. Mugyenyi, Philip Bejon, Kevin Marsh, James G. Beeson, Andrew M. Prentice, Hal Drakesmith, Thomas N. Williams

**Affiliations:** a Kenya Medical Research Institute (KEMRI) - Wellcome Trust Research Programme (KWTRP), PO Box 230-80108, Kilifi, Kenya; b Department of Paediatrics, Oxford University Hospitals, University of Oxford, Oxford, UK; c Oxford University Clinical Academic Graduate School, Oxford, UK; d Medical Research Unit (MRC) Human Immunology Unit, Weatherall Institute of Molecular Medicine, University of Oxford, Oxford University Hospitals, UK; e National Institute for Health Research Biomedical Research Centre Oxford, UK; f Burnet Institute, Melbourne, Victoria, Australia; g Department of Microbiology, Monash University, Victoria, Australia; h Medical Research Council (MRC) Unit, The Gambia; i Medical Research Council (MRC) International Nutrition Group, London School of Hygiene and Tropical Medicine, London, UK; j Department of Medicine, Imperial College, London, UK

**Keywords:** Malaria, Hepcidin, Iron deficiency, Children, Age, Africa

## Abstract

Both iron deficiency (ID) and malaria are common among African children. Studies show that the iron-regulatory hormone hepcidin is induced by malaria, but few studies have investigated this relationship longitudinally. We measured hepcidin concentrations, markers of iron status, and antibodies to malaria antigens during two cross-sectional surveys within a cohort of 324 Kenyan children ≤ 8 years old who were under intensive surveillance for malaria and other febrile illnesses. Hepcidin concentrations were the highest in the youngest, and female infants, declined rapidly in infancy and more gradually thereafter. Asymptomatic malaria and malaria antibody titres were positively associated with hepcidin concentrations. Recent episodes of febrile malaria were associated with high hepcidin concentrations that fell over time. Hepcidin concentrations were not associated with the subsequent risk of either malaria or other febrile illnesses. Given that iron absorption is impaired by hepcidin, our data suggest that asymptomatic and febrile malaria contribute to the high burden of ID seen in African children. Further, the effectiveness of iron supplementation may be sub-optimal in the presence of asymptomatic malaria. Thus, strategies to prevent and eliminate malaria may have the added benefit of addressing an important cause of ID for African children.

## Highlights

• Hepcidin fluctuates with age with the highest levels in female infants.
• Asymptomatic and febrile malaria increase hepcidin levels, but hepcidin does not predict the risk of malaria or febrile illness.
• Strategies to control malaria may have the added benefit of reducing iron deficiency in children

Iron deficiency and malaria are common among children living in Africa. Hepcidin is a hormone that inhibits dietary iron absorption. Hepcidin levels are increased during malaria infection and this protects against infection in mice. In Kenyan children we found that hepcidin levels varied by age and in infants by sex. Malaria increased hepcidin levels in well children and for a time after treatment in sick children. Hepcidin levels increased as antibodies to malaria increased, but hepcidin did not protect children from getting malaria. Our study suggests that malaria may be one of the causes of iron deficiency in African children.

## 1 Introduction

Malaria and iron deficiency (ID) are major public health problems for children living in sub-Saharan Africa. Malaria caused an estimated 437,000 deaths in young African children in 2013 (WHO, 2014) and > 70% of children have asymptomatic malaria in some malaria-endemic areas (Houngbedji et al., 2015), while ID is thought to impair cognitive development (Black et al., 2011) and is the leading cause of years lived with disability in sub-Saharan Africa (Vos et al., 2012). Hepcidin, the iron-regulatory hormone, may provide a critical link between malaria and ID. Hepcidin controls the absorption and distribution of iron (Ganz, 2013) and is thought to play a role in the innate immune response by restricting iron availability for pathogen growth (Ganz, 2009, Drakesmith and Prentice, 2012). The synthesis of hepcidin is regulated by diverse, often competing, physiological processes, including iron stores, inflammation and erythropoietic drive (Ganz, 2011, Atkinson et al., 2014). Malaria also alters hepcidin concentrations. Febrile malaria is associated with increased plasma concentrations (Howard et al., 2007, de Mast et al., 2009, Casals-Pascual et al., 2012, Ayoya et al., 2009), while severe and complicated malaria is associated with reduced plasma levels in African children (Casals-Pascual et al., 2012, Burte et al., 2013). Asymptomatic malaria also increased plasma levels in Indonesian school-age children (de Mast et al., 2010). In turn, we hypothesized that hepcidin may mediate the risk of malaria and other infections by restricting iron availability (Ganz, 2009, Drakesmith and Prentice, 2012). Intriguing data from mouse models suggest that hepcidin may play a critical role in host defence against malaria (Wang et al., 2011), malaria superinfection (Portugal et al., 2011), and bacterial infection (Arezes et al., 2015), but how this may work in children is not known. In the current study, our objectives were to assess the effect of a range of factors including age, gender and malaria on hepcidin concentrations and in turn to assess the effect of hepcidin concentrations on subsequent infectious risk in a longitudinal surveillance study of Kenyan children intensively monitored for malaria and other febrile illnesses.

## 2 Materials and Methods

### 2.1 Ethics Statement

Individual written informed consent was obtained from the parents of all study participants and ethical permission for the study was granted by the Kenya Medical Research Institute (KEMRI)/National Ethical Review Committee.

### 2.2 Participants and Procedures

**Fig. 1:**
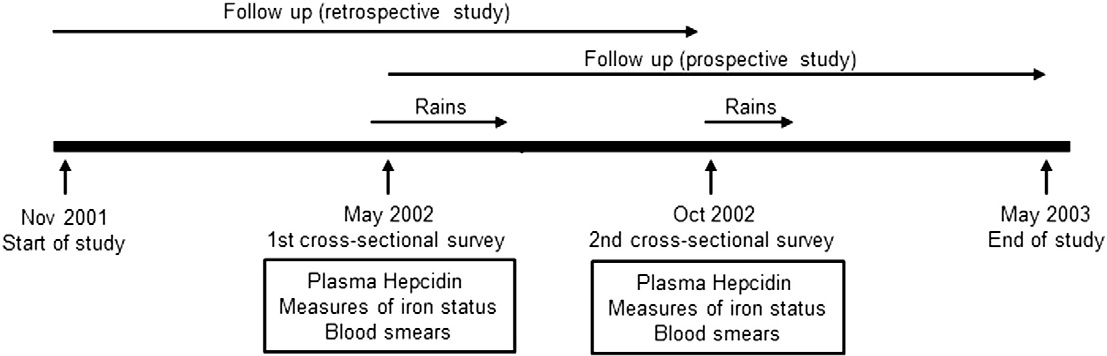
Study construction. A total of 324 children were recruited to the study; 245 contributed data to both the May and October surveys, 48 to the May survey only and 31 to the October survey only.

The current study was nested within an ongoing, longitudinal cohort study evaluating the history and acquisition of natural immunity to malaria in children living in Kilifi District on the Kenyan coast (Mwangi et al., 2005). The current study involving 324 children was conducted during an 18-month period between November 2001 and May 2003 and included all children < 8 years of age within the Ngerenya study area (Fig. 1). Participants were monitored for malaria and other diseases by weekly active surveillance as previously described (Mwangi et al., 2005). Two cross-sectional surveys were conducted at 6 and 12 months after the start of the study during which venous blood samples were collected. Children exited the study if informed consent was withdrawn or if they moved out of the study area for a period of > 2 months.

### 2.3 Laboratory Procedures

*Plasmodium falciparum* parasitaemia was determined as previously described (Nyakeriga et al., 2004). Haemoglobin typing (HbA and HbS) was by electrophoresis (Helena Laboratories, Beaumont, TX) while α-thalassemia genotyping was by PCR (Chong et al., 2000). Plasma concentrations of ferritin, soluble transferrin receptor (sTfR) and C-reactive protein (CRP) were determined as previously described (Atkinson et al., 2014, Nyakeriga et al., 2004). IgG antibodies against whole *P*. *falciparum* schizont extract and against the 3D7 allele of apical membrane antigen 1 (AMA1) and merozoite surface protein 2 (MSP2) were assayed by enzyme linked immunosorbent assay (ELISA) (Mugyenyi et al., 2013).

Plasma hepcidin was quantified by competitive ELISA (Hepcidin-25 (human) EIA Kit, Bachem) (Atkinson et al., 2014). Standards and samples were analyzed in duplicate or triplicate. Samples giving readings outside the standard linear region were repeated at appropriate dilutions. Readings with coefficient of variation > 10% were repeated. The lower limit of detection (LOD) of hepcidin was estimated at 0.08 ng/ml based on the hepcidin value corresponding to 3 standard deviations below the mean no hepcidin blank optical density at 450 nm; undiluted samples giving reading of < LOD were reported as LOD/2 = 0.04 ng/ml.

### 2.4 Case Definitions

Clinical malaria was defined as a fever (axillary temperature ≥ 37.5 °C) in conjunction with a positive blood smear for *P. falciparum* parasites at any density for children age < 1 year or at a density of > 2500 parasites/μl for children age ≥ 1 year (Mwangi et al., 2005). Asymptomatic malaria was defined during cross-sectional surveys as smear positive *P. falciparum* malaria in the absence of fever or other symptoms of clinical illness, while non-malarial fever was defined as a fever in conjunction with a negative malaria blood smear. Inflammation was defined as plasma CRP concentration of ≥ 5 mg/l (WHO, CDC, 2007). ID was defined as a ferritin concentration of < 12 μg/l, or < 30 μg/l in the presence of inflammation respectively (Atkinson et al., 2014, WHO/UNICEF/UNU, 2001). The ferritin index, a measure of bone marrow iron depletion, was defined as soluble transferrin receptor/log ferritin (Punnonen et al., 1997).

### 2.5 Statistical Analyses

All analyses were conducted using STATA v.12.0 (StataCorp. College Station, TX). Associations between hepcidin concentration (or other variables such as iron status) and independent parameters were evaluated using generalized estimating equation (GEE)-based linear regression models that included an exchangeable correlation structure and a robust variance estimator to account for correlation between measurements at two time points from the same child. Analyses were age-adjusted as appropriate. We did not restrict fitting independent parameters, such as age, to linear effects. We allowed for nonlinear effects by fitting and significance testing multivariable fractional polynomials with use of the Royston and Altman algorithm entering hepcidin concentration and other variables simultaneously in the model. This allowed the model to optimize the model fit using power and log functions to approximate the shape of the relationship of the parameter with hepcidin (Royston and Altman, 1994). The association between hepcidin concentration and the subsequent risk of clinical malaria or non-malarial fever was evaluated using Cox proportional hazards analysis during the 6-month period of monitoring after each cross-sectional survey. Therefore, each of the 324 children could contribute up to 2 periods of observation and the sandwich estimator was used to cluster analysis by individual (Armitage et al., 2001).

Multivariable models included covariates with a significance of p ≤ 0·1 in univariable models. We used p < 0·05 to interpret the findings in the final multivariable model. For clinical malaria hazards ratios were adjusted for age, ethnicity, sickle cell trait and period of monitoring and for non-malarial fever hazards ratios were adjusted for age in years and period of monitoring.

### 2.6 Role of the Funding Source

This work was funded by the Oxford University Clinical Academic Graduate School; The Academy of Medical Sciences with The Wellcome Trust, The British Heart Foundation, Arthritis Research UK (to SHA); a Beit Memorial Fellowship for Medical Research; an MRC New Investigator award; the National Institute for Health Research Oxford Biomedical Research Centre (to HD); a Senior Wellcome Trust Fellowship (grant number 091758 to TNW); a Senior Research Fellowship to JGB; and the European Union Framework Programme Seven European Virtual Institute of Malaria Research Consortium (grant number 242095 to TNW). The sponsors had no role in study design, data collection, data analyses, data interpretation, or writing of the report. The corresponding author had full access to all data in the study and the final responsibility for the decision to submit for publication.

## 3 Results

A total of 324 children were included in the study with an average followup time of 7·4 months. Table 1 describes the characteristics of the study population. Median age was 47·0 months at the mid-point of longitudinal follow-up (range; 4·9 to 97·1 months) and 54·6% were male. The overall geometric mean plasma hepcidin concentration was 2·45 ng/ml (95% CI; 2·08, 2·90; range; 0·04 to 176·56 ng/ml). ID and inflammation were common at 46% (262/572) and 18% (102/572) respectively. Asymptomatic malaria parasitaemia was also common: 12% (70/582) of routine blood smears were positive for *P*. *falciparum* with a mean parasite density of 909 parasites/μl (range; 40 to 380,000 parasites/μl). We found no significant association between geometric mean hepcidin concentrations and either sickle cell trait (for HbAA 2·48 ng/ml; 95% CI 2·08, 2·97; and for HbAS 2·15 ng/ml; 1·32, 3·50; p = 0·63) or α-thalassaemia (for αα/αα 2·64 ng/ml; 95% CI 1·96, 3·56; for − α/αα 2·31 ng/ml; 1·82, 2·93; p = 0·56 and for − α/− α 2·27 ng/ml; 1·48, 3·50; p = 0·61).

### 3.1 Hepcidin Concentration Varies by Age and in Infancy by Sex

**Fig. 2:**
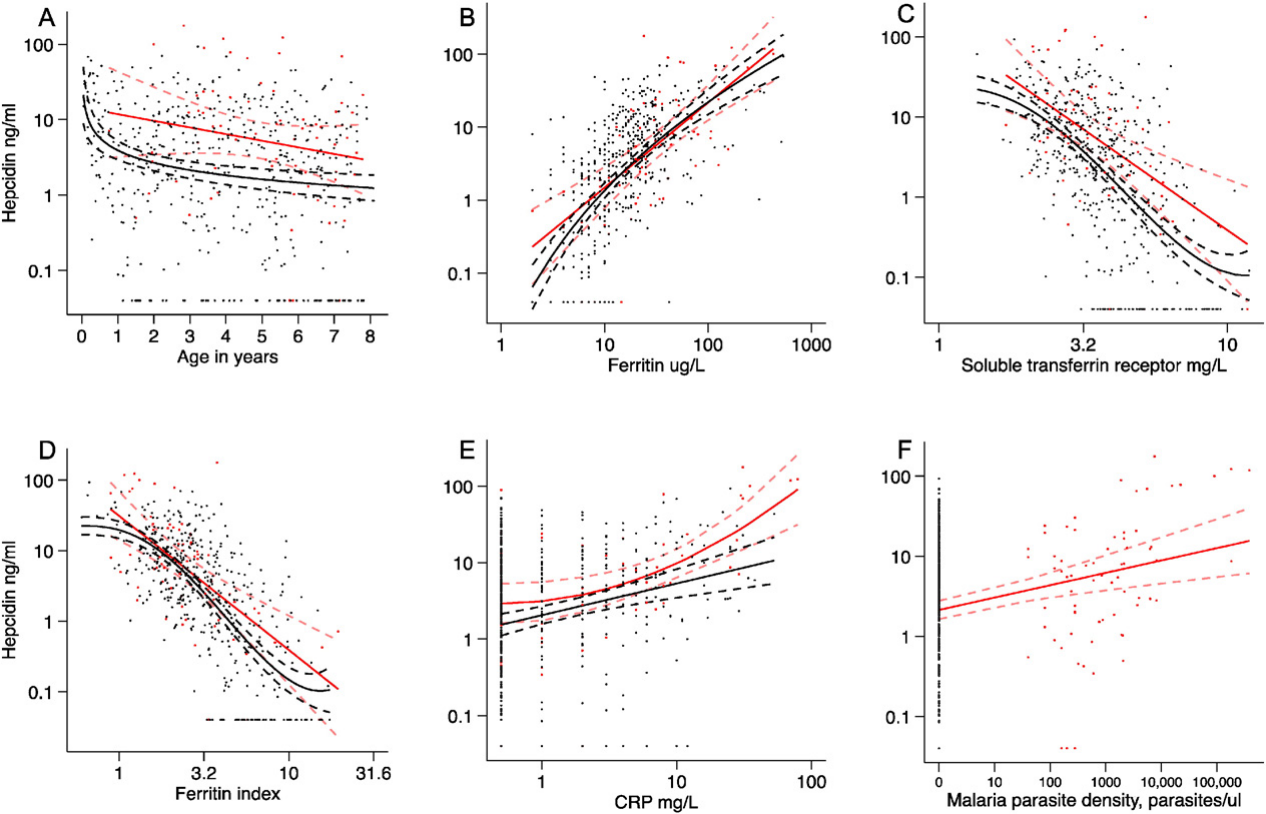
Multiple fractional polynomials of determinants of hepcidin concentrationby malaria parasitaemia. Scatter plot of log hepcidin concentration (y axis) against: A) age in years (x axis) with the fitted fractional polynomials: parasite positive (red): concentration = m_1_*(age in years) + c (n = 69); and parasite negative (black): concentration = m_1_*ln(age in years) + c (n = 494); B) Log ferritin (x axis) with the fitted fractional polynomials: parasite positive (red): concentration = m_1_*ln(ferritin) + c (n = 68); and parasite negative (black): concentration = m_1_*ln(ln(ferritin + c_1_)) + m_2_*(ln(ln(ferritin + c_1_)))^2^ + c_2_ (n = 489); C) Log sTfR (x axis) with the fitted fractional polynomials: parasite positive (red): concentration = m_1_*ln(sTfR) + c (n = 69); and parasite negative (black): concentration = m_1_*(ln sTfR)^3^ + m_2_*(ln sTfR)^3^*ln(ln sTfR) + c_1_ (n = 488); D) Log ferritin index (x axis) with the fitted fractional polynomials: parasite positive (red): concentration = m_1_*ln(ferritin index) + c (n = 68); and parasite negative (black): concentration = m_1_*(ln(ferritin index) + c_1_)^3^ + m_2_*(ln(ferritin index))^3^*ln(ln(ferritin index))^3^+ c_2_ (n = 475); E) Log CRP (x axis) with the fitted fractional polynomials: parasite positive (red): concentration = m_1_*(ln(CRP) + c_1_)^2^ + c_2_ (n = 68); and parasite negative (black): concentration = m_1_*ln(CRP) + c (n = 489); F) *P*. *falciparum* parasite density, parasites/μl (x axis) with the fitted fractional polynomial: Concentration = m_1_*ln(parasite density) + c (n = 69). Dotted lines indicate 95% confidence intervals. (For interpretation of the references to colour in this figure legend, the reader is referred to the web version of this article.)

**Fig. 3:**
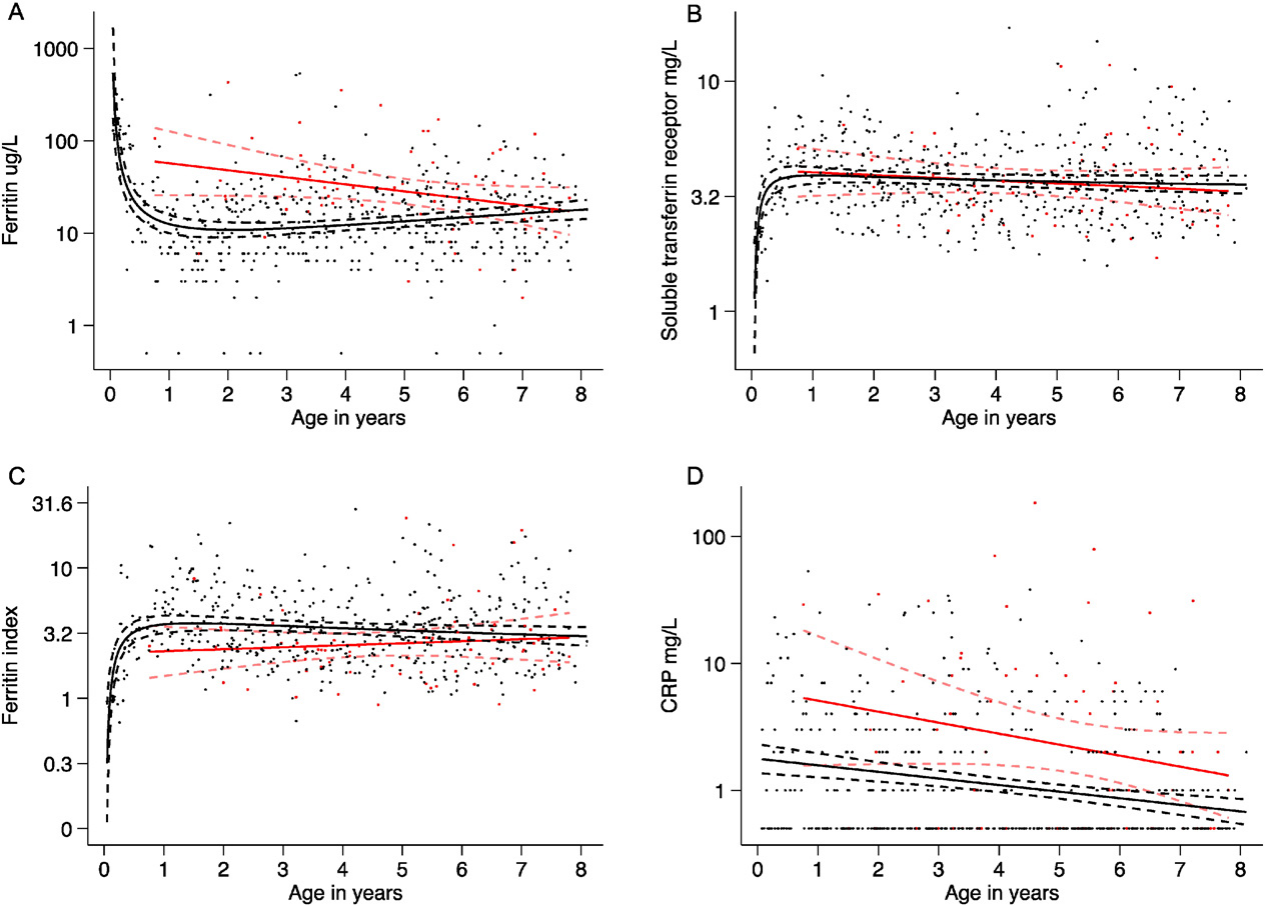
Multiple fractional polynomials of the age profile of iron status and inflammation by malaria parasitaemia. Scatterplots of age in years (x axis) against A) log ferritin with the fitted fractional polynomials: for parasite positive (red): concentration = m_1_*(age in years) + c (n = 69); and parasite negative (black): concentration = m_1_*ln(age in years) + m_2_*(ln(age in years))^2^ + c (n = 496); B) log soluble transferrin receptor with the fitted fractional polynomials: for parasite positive (red): concentration = m_1_*(age in years) + c (n = 70); and parasite negative (black): concentration = m_1_*(age in years)^− 0.5^ + m_2_*(age in years)^− 0.5^ *ln(age in years) + c (n = 498); C) log ferritin index with the fitted fractional polynomials for parasite positive (red): concentration = m_1_*(age in years) + c (n = 69); and parasite negative (black): concentration = m_1_*(age in years)^− 0.5^ + m_2_*ln(age in years) + c (n = 481); and D) log CRP with the fitted fractional polynomials: for parasite positive (red): concentration = m_1_*(age in years) + c (n = 69); and parasite negative (black): concentration = m_1_*(age in years) + c (n = 496). Dotted lines indicate 95% confidence intervals. (For interpretation of the references to colour in this figure legend, the reader is referred to the web version of this article.)

Plasma hepcidin concentration varied markedly by age (ß = − 0·1; − 0·06, − 0·13; p < 0·0005 in a GEE-based model), while the best-fit fractional polynomial for the age profile of hepcidin suggested that levels were the highest in the youngest children, decreased very rapidly in infancy and then declined slowly in childhood (Fig. 2A). These findings may be explained by age-related differences in the strength of stimuli determining hepcidin expression. The best-fit for the age profile of ferritin suggested that levels fell very rapidly in infancy reaching a nadir between 1 and 2 years of age and then gradually increased. Soluble TfR levels and the ferritin index increased sharply in infancy, while the age profile of CRP suggested a simple linear decrease in inflammation with increasing age (Fig. 3A–D). The prevalence of asymptomatic malaria parasitaemia increased with increasing age (Table 2). We found no overall difference in hepcidin concentrations by sex in all age groups combined (2·75 ng/ml; 2·11, 3·57 for females vs. 2·24; 1·80, 2·78 for males; p = 0·40 in an age-adjusted model), however female infants had markedly higher concentrations (12·32 ng/ml; 95% CI 7·54, 20·12) compared to male infants (5·79 ng/ml; 3·44, 9·76; p = 0 · 009).

### 3.2 Hepcidin Concentration is Influenced by Iron Stores, Erythropoiesis and Inflammation

Ferritin, sTfR, and CRP levels explained 37.5%, 37.3%, and 10.2% of the variance in hepcidin concentrations respectively (p < 0.0005 for each). Hepcidin concentrations were very low in children with ID (0·71 ng/ml; 95% CI 0·55, 0·92) in comparison to those without (6·64 ng/ml; 95% CI 5·71, 7·70; p < 0·0005). The best-fit fractional polynomials suggested that hepcidin concentrations increased with increasing ferritin and CRP and decreased with increasing sTfR and ferritin index (Fig. 2B–E). Inflammation (CRP ≥ 5 mg/l) was associated with increased hepcidin concentrations (7·57 ng/ml; 95% CI 5·56, 10·31 compared to 1·91; 1·58, 2·30; p < 0·0005, in those without inflammation).

### 3.3 Hepcidin Concentration is Positively Associated with Asymptomatic Malaria

**Fig. 4:**
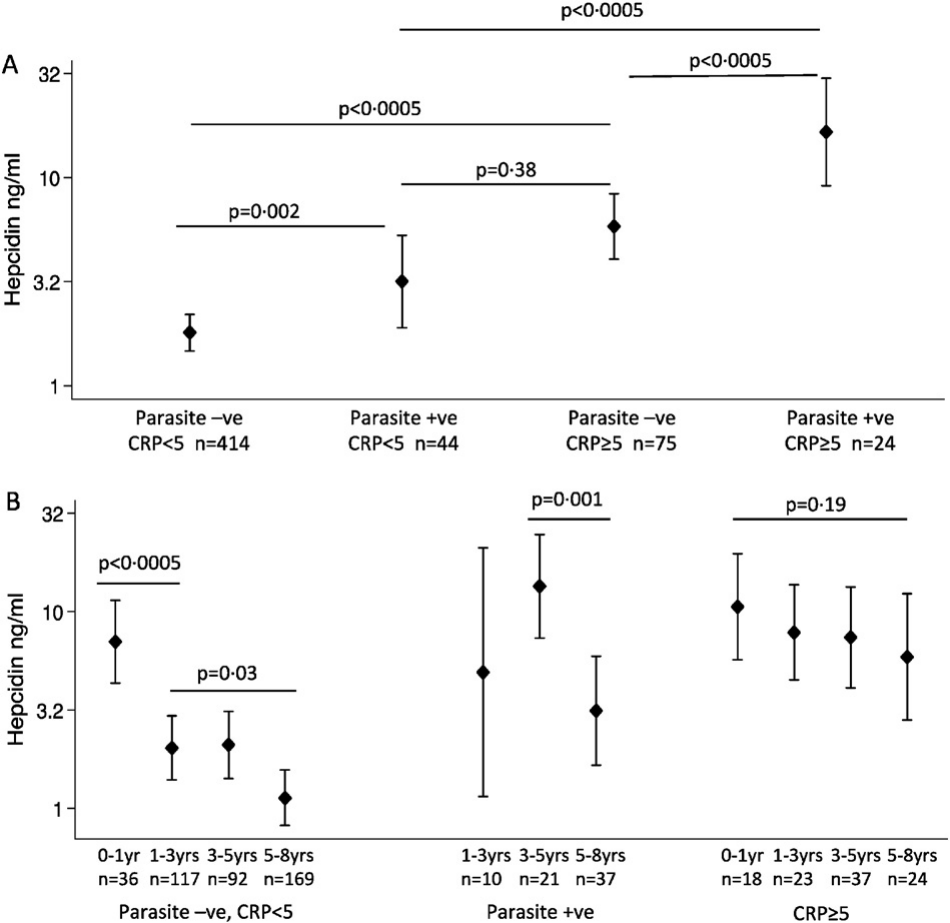
Malaria parasitaemia and inflammation influence hepcidin concentrations. Geometric mean hepcidin concentrations (and 95% confidence intervals) by malaria parasitaemia and inflammation for: A) all children and B) according to age group. P values are derived from GEE-based regression models, and analyses that included all ages were adjusted for age.

Hepcidin concentrations were more than doubled in individuals with asymptomatic *P*. *falciparum* parasitaemia (5·29 ng/ml; 3·39, 8·25 compared to 2·18 ng/ml; 1·82, 2·61; p < 0·0005 in aparasitaemic individuals) and malaria parasitaemia explained 7% of the variation in hepcidin (p < 0·0005). Parasite density, ID, inflammation, and age modified the effects of parasitaemia on hepcidin (Figs. 2F, 4). Among parasitized children those with ID had markedly lower hepcidin concentrations compared to those without (1·41 ng/ml; 0·49, 4·0 vs. 8·14 ng/ml; 5·26, 12·6; p < 0·0005). Moreover, parasitaemia increased hepcidin concentrations both in the absence and presence of inflammation (Fig. 4A). Hepcidin concentrations were higher among younger parasitaemic children than older (13·47 ng/ml; for 3–5 years old compared to 3·14 ng/ml for 5–8 years old; β -0·64; − 0·27, − 1·0; p = 0 · 001, Figs. 2A and 4B). Similarly, ferritin levels were higher in younger parasitaemic children (Fig. 3A), although CRP and parasite density did not differ significantly between age groups (Table 2).

### 3.4 Hepcidin Concentration is Positively Associated with Antibodies to *P. falciparum* Antigens

We then assessed whether hepcidin concentrations correlated with antibodies to *P*. *falciparum* antigens. In age-adjusted GEE-based models hepcidin concentrations were positively associated with antibody titres to schizont extract, AMA1, and MSP2 (β 0·41; 0·29, 0·53; p < 0·0005; β 0·13; 0·02, 0·23; p = 0·02, and β 0·23; 0·13, 0·33; p < 0·0005 respectively). We found a significant interaction between age and antibody titres to AMA1 in predicting hepcidin concentrations (p = 0·01) and a trend towards an interaction with MSP2 (p = 0·08), so that in younger children a smaller unit change in AMA1 or MSP2 was associated with a much larger unit change in hepcidin compared with older children. We found no interaction between age and antibody titres to schizont extract in predicting hepcidin concentrations.

### 3.5 Recent Malaria, but not Non-malarial Fever, Alters Hepcidin Concentrations

**Fig. 5:**
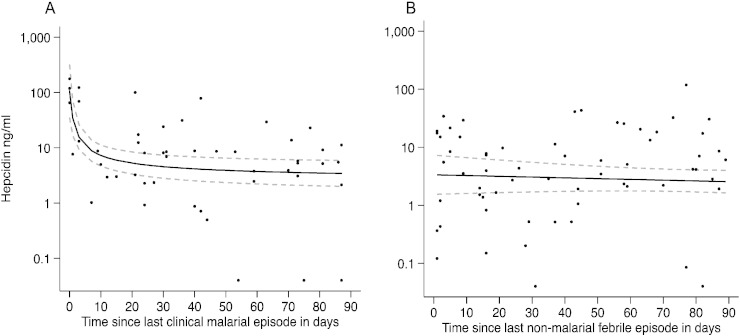
Hepcidin concentrations following clinical malaria and non-malarial febrile illness. Scatterplots of log hepcidin concentration (y axis) against time since A) last clinical malarial episode (x axis) with fitted fractional polynomial, concentrations = m_1_*(time since last clinical malarial episode) + c and B) last febrile non-malarial episode with fitted fractional polynomial, concentrations = m_1_*(time since last febrile non-malarial episode) + c. Time was restricted to a 3 month period prior to hepcidin measurement.

We next evaluated whether hepcidin concentrations varied with time after a clinical malaria or non-malarial fever episode. The best-fit fractional polynomial of the time profile of hepcidin after treatment for clinical malaria suggested that hepcidin concentrations declined steeply in the first week, then more slowly over the subsequent weeks (Fig. 5A). However, we found no significant difference in hepcidin concentration attributable to non-malarial febrile illnesses (Fig. 5B).

### 3.6 Hepcidin Concentration does not Predict the Subsequent Risk of Malaria or Non-malarial Fever

**Fig. 6:**
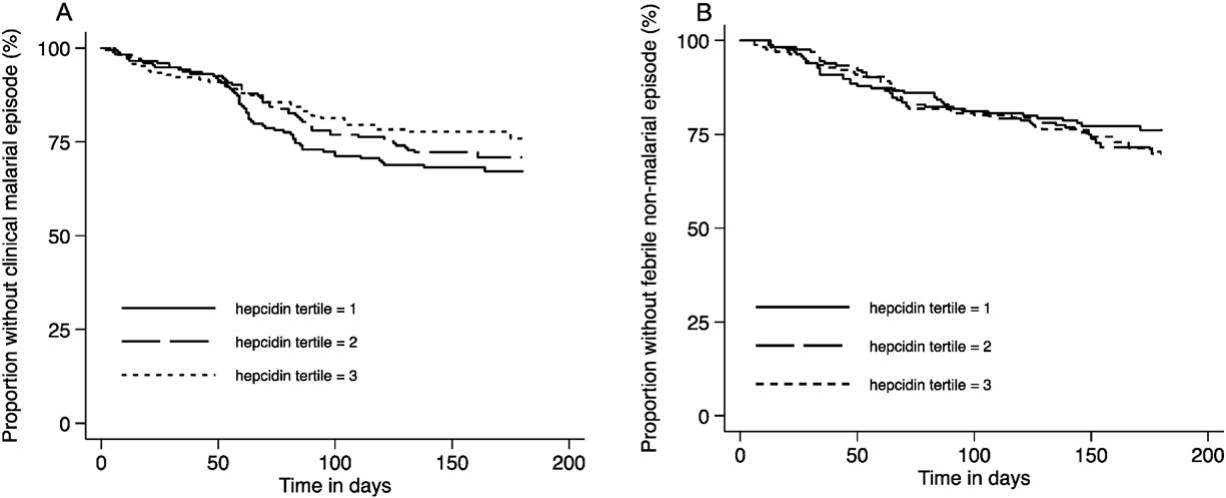
Kaplan–Meier curves of A) time to first clinical malarial episode according to tertile of hepcidin concentration (p = 0.43 for 1st tertile vs. 2nd tertile and p = 0.07 for 1st tertile vs. 3rd tertile); and B) time to first non-malarial fever episode according to tertile of hepcidin concentration (p = 0.50 for 1st tertile vs. 2nd tertile and p = 0.18 for 1st tertile vs. 3rd tertile). The range of hepcidin concentration for each hepcidin tertile was: 0·04–1·60 ng/ml for the 1st tertile; 1·61–7·49 ng/ml for the 2nd tertile; and 7·50–122·47 ng/ml for the 3rd tertile. P values are derived from Cox regression models.

Finally, we tested the hypothesis that hepcidin concentrations influence the subsequent risk of malaria and non-malarial fever. Overall, we observed 148 first or only episodes of clinical malaria and 130 first or only episodes of non-malarial fever during 2402 months of monitoring. Survival plots for clinical malaria and non-malarial fever by hepcidin tertile are shown in Fig. 6. In univariable Cox analyses we observed a trend towards a reduced risk of clinical malaria with higher hepcidin concentrations (HR 0.85; 0.71, 1.01; p = 0·07), an effect that was lost on adjustment for other variables (adjusted HR 1.08; 0.90, 1.30; p = 0.41, Table 3). We similarly found no association between hepcidin and the subsequent risk of non-malarial febrile illness (Table 3).

## 4 Discussion

Both malaria and ID are important public health problems in African children. In the current study 12% of children were parasitaemic and 46% had ID. Hepcidin concentrations fell rapidly in infancy and then more slowly with increasing age, while female infants had higher concentrations than males. We found that asymptomatic malaria was associated with significantly elevated hepcidin concentrations, which were proportional to parasite density and modified by age, inflammation, and the presence of ID. Furthermore, hepcidin was positively associated with antibody titres to *P*. *falciparum* antigens. Concentrations fell rapidly and then slowly after treatment of clinical malaria, but were not altered by non-malarial febrile illnesses. Nevertheless, hepcidin concentrations did not predict the subsequent risk of malaria or other febrile illnesses.

We found a non-linear association between age and hepcidin. In agreement with a study in 3–12-month old Zimbabwean infants (Mupfudze et al., 2014), hepcidin concentrations were the highest in the youngest children and decreased dramatically in infancy (Fig. 2A). We add to this that hepcidin concentrations are the highest in the first 3 months of life. However, in contrast to European studies, which showed either no change or an increase in hepcidin with age (Sdogou et al., 2015, Cangemi et al., 2013), we found a slow decline in hepcidin with increasing age. These findings might be explained by age-specific differences in the strength of hepcidin stimuli (such as iron stores, erythropoietic drive, and inflammation) (Fig. 3), and we hypothesize that different environmental conditions may influence hepcidin signalling at varying ages in European children. In agreement with a study in Kenyan infants (Jaeggi et al., 2013), we found that hepcidin concentrations were about twice as high in female infants compared to males.

Ferritin, sTfR and CRP explained 37·5%, 37·3%, and 10·2% of the variance in hepcidin respectively (p < 0·0005 for each). In agreement with a study in Indonesian school children (de Mast et al., 2010), asymptomatic malaria was also associated with an increase in plasma hepcidin (explaining 7% of variance; p < 0·0005), both in the presence and absence of inflammation, suggesting that malaria regulates hepcidin via non-inflammatory, as well as inflammatory pathways (Armitage et al., 2009). We add that hepcidin levels are proportional to parasite density in asymptomatic infection, as observed in febrile malaria (Howard et al., 2007, Casals-Pascual et al., 2012), but not in severe infection (Burte et al., 2013). Hepcidin concentrations were markedly higher in younger compared to older parasitized children, suggesting that iron absorption is more likely to be impaired in younger children. Finally, among children with parasitaemia, hepcidin levels were markedly lower in those with ID than those without ID, suggesting that malaria-mediated up-regulating stimuli may be overruled by iron demand and erythropoietic stimuli down-regulating hepcidin synthesis. Thus, children with ID and parasitaemia might be in danger of receiving iron if hepcidin-guided supplementation was implemented without malaria screening.

Antibodies to malaria antigens are sensitive biomarkers of population-level malaria exposure and may be useful surveillance tools for malaria control and elimination (Elliott et al., 2014). We investigated whether malaria antibodies might reflect malaria-induced hepcidin levels. We found that plasma hepcidin concentrations were strongly positively associated with antibodies to *P*. *falciparum* antigens in age-adjusted models. For AMA1 and MSP2 we found interactions between age and antibody titres so that in younger children a small increase in antibody titres was associated with a large increase in hepcidin concentrations, while in older children increased antibody titres had little effect on hepcidin. This agrees with our study showing that these antibodies act as measures of exposure in younger children with lower immunity and as measures of protective immunity in older children with higher antibody titres (Stanisic et al., 2015). By contrast antibodies to schizont extract were strongly positively associated with hepcidin concentrations regardless of age, which might be explained by their shorter half-life and high rates of sero-reversion at all ages (Ondigo et al., 2014). Taken together, these data suggest that sero-surveillance tools may be of use to identify population-level malaria-induced hepcidin, particularly in younger children, with relevance to programmes to restore healthy iron status.

We next evaluated the profile of hepcidin concentrations over a 3-month period after exposure to and treatment of febrile clinical malaria. In agreement with other studies in African children we found that concentrations were initially very high (Howard et al., 2007, de Mast et al., 2009, Casals-Pascual et al., 2012, Burte et al., 2013); but fell very rapidly in the first week following treatment of febrile malaria and more slowly in the subsequent weeks (Fig. 5A) (de Mast et al., 2009, Casals-Pascual et al., 2012). Contrary to expectations, non-malarial febrile illnesses were not significantly associated with hepcidin concentrations (Fig. 5B), although the fact that diagnoses were not assigned to non-malarial febrile illnesses is a limitation of the study. However, in agreement with this finding, children with both inflammation and malaria had markedly higher hepcidin concentrations than those with inflammation alone (p < 0.0005, Fig. 4A), suggesting that malaria may have a more pronounced effect on hepcidin synthesis than other febrile illnesses.

Finally we hypothesized that hepcidin concentrations might influence the subsequent risk of malaria. Intriguing data from mouse studies suggest that hepcidin might play a role in modulating clinical malaria (Wang et al., 2011, Portugal et al., 2011), but we are not aware of any previous studies that have investigated this possibility in humans. After adjustment for potential confounders, we found no significant association between hepcidin and the subsequent risk of either *P*. *falciparum* malaria or non-malarial fever. A number of possible explanations can be put forward for this negative finding. It is possible that, in contrast to mouse studies (Wang et al., 2011), hepcidin concentrations are not associated with severity of malaria or other infections in humans, perhaps due to hepcidin-independent iron restriction (Guida et al., 2015). However, if they are, we may have failed to detect an effect for a number of reasons. Firstly, baseline hepcidin may not influence hepcidin at the time of, or just prior to, acute malaria infection and a limitation of our study is that hepcidin was not measured routinely at the time of acute malaria. Second, given that hepcidin is controlled by multiple competing stimuli (Huang et al., 2009), hepcidin might only influence the subsequent risk of infection when very strong down-regulatory signals from ID and erythropoietic drive overwhelm weaker up-regulatory signals from malaria and other infections (Casals-Pascual et al., 2012, Jonker et al., 2013). However, the study cohort consisted of ‘healthy’ community-based children. Finally, there may also be counter-balancing effects, for example a reduced risk of malaria due to ID (Nyakeriga et al., 2004, Gwamaka et al., 2012) may counter a protective effect of higher hepcidin concentrations: all questions for future studies.

In conclusion, we have shown that asymptomatic and recent febrile malaria both significantly increase hepcidin in a population of children where ID is an important cause of morbidity. Given that dietary iron absorption is impaired by elevated hepcidin (Cercamondi et al., 2010, Prentice et al., 2012, Glinz et al., 2015), our data suggest that asymptomatic and febrile malaria contribute to the high prevalence of ID in African children. Unless malaria episodes are controlled, iron supplementation may be ineffective because of hepcidin-mediated poor iron absorption. Strategies to prevent and eliminate malaria may therefore have the added benefit of addressing an important cause of ID for children living in sub-Saharan Africa.

## Abbreviations

ID: iron deficiency
GEE: generalized estimating equation
AMA1: apical membrane antigen 1
MSP2: merozoite surface protein 2
CRP: C-reactive protein
HR: hazard ratio

## Author contributions

SHA, HD, AMP, JGB, TNW, SK, SMU, CKM, AEA, and PB wrote the manuscript. SHA, HD, AEA, and TNW conceived the study. All authors contributed to the design of the study. SK, SMU, and CKM performed laboratory analyses and data collection, SK performed hepcidin and iron measurements and SHA and PB performed all statistical analyses.

## Declaration of interests

All authors declare no conflicts of interest.

## Figures and Tables

**Table 1:** Characteristics of study population (n = 324).

Characteristics	
Median age, mths^a^ (range)	47·0 (4·9, 97·1)
Male sex, n (%)	177 (54·6)
Haemoglobin AS, n (%)^b^	45 (14·0)
α^+^thalassemia, n (%)^c^	
αα/αα	95 (30·3)
αα/− α	162 (51·8)
− α/− α	56 (17·9)
Ethnic group, n (%)^d^	
Giriama	279 (86·1)
Chonyi	28 (8·6)
Kauma	17 (5·3)

a Defined as age at the mid-point of individual longitudinal follow up;

b 2 missing haemoglobin S type;

c 11 missing α^+^thalassaemia genotypes;

d Subgroups of the Mijikenda ethnic group.

**Table 2:** Effects of age on hepcidin, inflammation and iron status by malaria parasitaemia.

		Age, years			
		0–1 year	1–3 years	3–5 years	5–8 years
Number, (%)		63 (10·6)	161 (27·1)	142 (24·0)	227 (38·3)
Malaria parasitaemia, n (%)		1 /62 (1·6)	10/155 (6·5)	21/141 (14·9)	38/224 (17·0)
Parasite density, parasites/μL		120	1660 (327, 8406)	984 (380, 2545)	783 (407, 1505)
Hepcidin, ng/ml	All	8·39 (5·85, 12·05)	2·49 (1·82, 3·41)	3·30 (2·39, 4·55)	1·47 (1·11, 1·95)
Parasite + ve	7·05	4·92 (1·15, 21·15)	13·47 (7·35, 24·70)	3·14 (1·66, 5·95)
Parasite -ve	8·42 (5·83, 12·17)	2·31 (1·66, 3·21)	2·56 (1·81, 3·64)	1·25 (0·91, 1·70)
CRP, mg/L	All	2·08 (1·44, 3·02)	1·29 (1·07, 1·57)	1·55 (1·22, 1·96)	0·89 (0·79, 1·02)
Parasite + ve	29	2·48 (0·75, 8·14)	3·28 (1·45, 7·44)	1·74 (1·08, 2·78)
Parasite -ve	1·99 (1·38, 2·87)	1·20 (1·0, 1·46)	1·32 (1·05, 1·67)	0·79 (0·70, 0·89)
Ferritin, μg/L	All	27·7 (19·5, 39·4)	11·0 (9·4, 13·0)	18·1 (15·3, 21·4)	14·8 (13·0, 16·9)
Parasite + ve	106	30·4 (12·8, 72·3)	42·6 (29·3, 62·0)	21·5 (15·0, 30·8)
Parasite -ve	27·0 (19·0, 38·6)	10·2 (8·7, 12·0)	15·4 (12·9, 18·3)	13·9 (12·1, 15·9)
sTfR, mg/L	All	3·44 (3·09, 3.83)	3·92 (3·72, 4·13)	3·38 (3·19, 3·58)	3·74 (3·53, 3·96)
Parasite + ve	5·09	4·49 (3·61, 5·58)	3·02 (2·74, 3·32)	3·71 (3·19, 4·32)
Parasite -ve	3·42 (3·07, 3·81)	3·88 (3·67, 4·11)	3·46 (3·24, 3·69)	3·74 (3·51, 3·98)
Ferritin index	All	2·54 (2·08, 3·09)	3·85 (3·48, 4·25)	2·82 (2·55, 3·11)	3·29 (3·00, 3·61)
Parasite + ve	2·51	3·19 (2·05, 4·97)	1·89 (1·63, 2·19)	3·04 (2·33, 3·97)
Parasite -ve	2·54 (2·08, 3·10)	3·90 (3·51, 4·34)	3·05 (2·73, 3·40)	3·35 (3·04, 3·69)

Abbreviations: CRP, C-reactive protein; sTfR, soluble transferrin receptors. Parasite + ve indicates the presence of malaria parasites on routine blood smear. Unless otherwise indicated numbers are geometric means with 95% confidence intervals in brackets.

**Table 3:** Cox regression models for risk of clinical malaria and non-malarial fever by hepcidin concentrations.

Clinical malaria^a^	HR (95% CI)	p	Adjusted HR (95% CI)	p
Log Hepcidin (ng/ml)	0·85 (0·71, 1·01)	0·07	1·08 (0·90, 1·30)	0·41
Hepcidin tertiles				
Hepcidin tertile 1	Reference	–	Reference	–
Hepcidin tertile 2	0·85 (0·58, 1·26)	0·43	1·07 (0·72, 1·58)	0·75
Hepcidin tertile 3	0·68 (0·45, 1·04)	0·07	1·00 (0·63, 1·58)	1·00

Non-malarial fever^b^	HR (95% CI)	p	Adjusted HR (95% CI)	p

Log hepcidin (ng/ml)	1·13 (0·91, 1·40)	0·25	1·02 (0·81, 1·27)	0·88
Hepcidin tertiles				
Hepcidin tertile 1	Reference	–	Reference	–
Hepcidin tertile 2	1·16 (0·75, 1·79)	0·50	1·05 (0·67, 1·63)	0·84
Hepcidin tertile 3	1·34 (0·87, 2·05)	0·18	1·12 (0·72, 1·74)	0·61

Abbreviations: HR, hazard ratio; CI, confidence interval. HRs and 95% CIs are shown for each log fold increase in hepcidin level and by hepcidin tertiles (tertile 1 = 0·04–1·60 ng/ml; tertile 2 = 1·61–7·49 ng/ml; tertile 3 = 7·50–122·47 ng/ml).

a Defined as a fever (axillary temperature ≥ 37·5 °C) in conjunction with a positive blood film at any density for children age < 1 year or at a density of > 2500 parasites/μl for children aged ≥ 1 year (Mwangi et al., 2005). For clinical malaria HRs were adjusted for age in years, ethnicity, sickle cell trait, and period of monitoring.

b Defined as a fever in conjunction with a negative blood film. For non-malarial fever HRs were adjusted for age in years and period of monitoring.
